# Development of Nomograms to Predict the Probability of Recurrence at Specific Sites in Patients with Cutaneous Melanoma

**DOI:** 10.3390/cancers17183080

**Published:** 2025-09-21

**Authors:** Eszter Anna Janka, Imre Lőrinc Szabó, Tünde Toka-Farkas, Lilla Soltész, Zita Szentkereszty-Kovács, Beatrix Ványai, Tünde Várvölgyi, Anikó Kapitány, Andrea Szegedi, Gabriella Emri

**Affiliations:** 1 Department of Dermatology, MTA Centre of Excellence, Faculty of Medicine, University of Debrecen, 4032 Debrecen, Hungary; janka.eszter@med.unideb.hu (E.A.J.);; 2 HUN-REN-UD Allergology Research Group, University of Debrecen, 4032 Debrecen, Hungary

**Keywords:** melanoma, prognostic factors, Cox proportional hazard models, nomograms, ROC curve

## Abstract

Background: Risk assessment models are increasingly being used in oncology to improve therapeutic and follow-up decisions for individual patients. Methods: In our study, we used a university hospital registry database containing data on patients diagnosed with invasive cutaneous melanoma between 2000 and 2019 (training cohort: N = 1402; validation cohort: N = 601). Using multivariate Cox regression models, we identified clinicopathological variables that are independent risk factors for melanoma recurrence at specific sites. We then constructed nomograms to predict the probability of recurrence at 3, 5, and 10 years. Results: Age, sex, primary tumor location, histological subtype, Clark invasion level and AJCC pT category were independent prognostic factors for melanoma recurrence in regional lymph nodes. Age, sex, primary tumor location, Clark level of invasion, AJCC pT stage and regional lymph node metastasis were risk factors for skin/soft tissue (including muscle)/non-regional lymph node metastases. We found that AJCC pT category and sex were also independent prognostic factors for melanoma recurrence in the lung, visceral sites, and brain. Furthermore, the nomogram predicting recurrence in the lung and visceral sites incorporated the presence of regional lymph node and skin/soft tissue/non-regional lymph node metastases. ROC curves showed good performance of the nomograms in both the training and validation cohorts. The calibration curve showed a good fit. Conclusion: Our results support the high prognostic value of AJCC pT stage and patient sex, which remained consistent across all melanoma stages, and demonstrate the feasibility of creating nomogram models to predict recurrence risk in melanoma patients.

## Simple Summary

The risk of recurrence of cutaneous melanoma after surgical removal of the primary tumor is high and varies depending on the location of the recurrence. We analyzed clinical and pathological data from approximately 2000 patients with invasive cutaneous melanoma to identify independent prognostic factors that may be useful in predicting the pattern of recurrence. We have developed nomograms to determine the 3, 5, and 10-year probability of recurrence at specific locations. The nomogram models showed good accuracy in predicting recurrence in (1) lymph nodes, (2) skin, soft tissues (including muscle), and/or non-regional lymph nodes, (3) lung, (4) visceral sites, and (5) brain. Of note, the AJCC 8th edition pT stage and patient sex were independent risk factors for melanoma recurrence at any sites. These nomograms may help clinicians assess individualized recurrence risk and tailor follow-up, as well as make more informed decisions about the treatment of melanoma patients.

## 1. Introduction

Cutaneous melanoma of the skin has attracted the attention of the medical community due to its increasing incidence, poor prognosis, and recent advances in therapy [1,2,3]. Ways of reducing melanoma-related morbidity and mortality include improving primary prevention, early detection and primary care, appropriate follow-up methods, and effective treatment for patients at high risk of metastasis and those with metastasis [4,5,6]. Prognostic considerations are highly relevant to the proper management of patients with melanoma, with risk prediction models remaining a research focus [7,8,9,10,11,12]. Although molecular prognostic factors such as germline and somatic driver mutations, gene expression profiles, epigenetic alterations, and immunological characteristics have been extensively studied, the complex interplay between these factors poses challenges for interpreting results [2,4,5,8,13,14]. In addition, the requirement for appropriate tissue samples and molecular assays to assess these markers limits their clinical utility [2]. Clinicopathological factors are readily available and their systematic evaluation within an evidence-based framework can provide clinicians with a greater degree of confidence in formulating patient management recommendations [4,5]. In order to determine whether the prognosis for melanoma patients can be improved by taking into account clinicopathological factors beyond the American Joint Committee on Cancer’s 8th edition TNM classification, we require reliable clinical data on a sufficient number of patients, as well as an evaluation of the data using appropriate multivariable statistical models [10,11,15]. The individual and combined effects of risk factors on time to disease recurrence and death can be assessed using a Cox regression model, which provides an estimation of the relative risk of a risk factor’s presence compared to its absence while adjusting for other risk factors [16]. The present study aims to contribute data collected from a Central European melanoma patient population to research on prognostic factors in melanoma. To increase the clinical impact, nomograms incorporating multiple risk factors were constructed to provide individual risk predictions for disease recurrence [17].

## 2. Materials and Methods

### 2.1. Study Population

This retrospective study included a total of 2003 patients diagnosed with invasive cutaneous melanoma between 2000 and 2019, who were followed up until 14 June 2025 or the date of death. The melanoma register data were collected from the integrated hospital information system used at the University of Debrecen (MedSolution and UDMed). The inclusion criterion was a melanoma diagnosis between 2000 and 2019. The exclusion criterion was the absence of clinicopathological and follow-up data for statistical analysis. The study was approved by the Medical Research Council Ethics Committee (certificate number IV/1711-4/2021/EKU).

The register included the following patient data: age; sex; histological subtype of primary melanoma; Breslow tumor thickness; ulceration status; localization; Clark invasion level; and primary tumor (pT) stage, according to the 8th edition of the American Joint Committee on Cancer (AJCC) TNM classification for melanoma [18]. Furthermore, we recorded the time and type of any metastases that developed from primary tumor diagnosis until the end of the follow-up period. Melanoma recurrences were identified through histopathological or imaging examinations (computed tomography (CT), magnetic resonance imaging (MRI) or positron emission tomography with 2-deoxy-2-[fluoro-18]fluoro-D-glucose integrated with computed tomography (18F-FDG PET/CT)), in accordance with routine practice.

### 2.2. Statistical Analysis

To validate our nomogram models, we used R statistical software to randomly divide our database into training and validation sets at a ratio of 7:3 (training cohort: N = 1402; validation cohort: N = 601). Chi-squared tests were used to analyze the categorical variables.

Recurrence-free survival (RFS) and overall survival (OS) were assessed using the Kaplan–Meier estimator, and survival probability comparisons were performed using a two-sided log-rank test. RFS was calculated by taking into account the time from diagnosis of the primary tumor to recurrence or the final follow-up. OS was calculated by taking into account the time from diagnosis of the primary tumor or first recurrence to the last follow-up or death. For occult melanoma (the AJCC 8th edition T0 category), follow-up was performed from the time of metastasis diagnosis. The median survival time (in years) is presented with a 95% confidence interval (95% CI). Univariate and multivariate hazard ratios (HR) were calculated with their respective 95% upper and lower confidence intervals using Cox regression analysis [19]. The significant independent prognostic factors for melanoma recurrence in (1) lymph nodes, (2) the skin and soft tissue (including muscle) and non-regional lymph nodes, (3) the lung, (4) non-central nervous system visceral sites and (5) the brain, as obtained during multivariate Cox regression, formed the basis for the nomograms. Accordingly, we created five nomograms to predict risk of 3-year, 5-year and 10-year recurrence at specific sites.

During the nomogram validation, the discriminatory ability of the model was evaluated based on the area under the receiver operating characteristic (ROC) curve (AUC) and the C-index. Calibration plots were created to evaluate predictive accuracy using the Bootstrap method with 1000 resamples. Decision curve analysis (DCA) was used to determine the net benefit of the model for patients.

The significance level was *p* < 0.05 in all cases. Statistical analyses were performed using IBM SPSS Statistics for Windows, version 25.0 (IBM Corp., Armonk, NY, USA), and R software, version 4.5.1 (R Foundation for Statistical Computing, Vienna, Austria). A biostatistician, E.A. Janka, supervised the statistical analysis.

## 3. Results

### 3.1. Patient and Disease Characteristics

**Figure 1:**
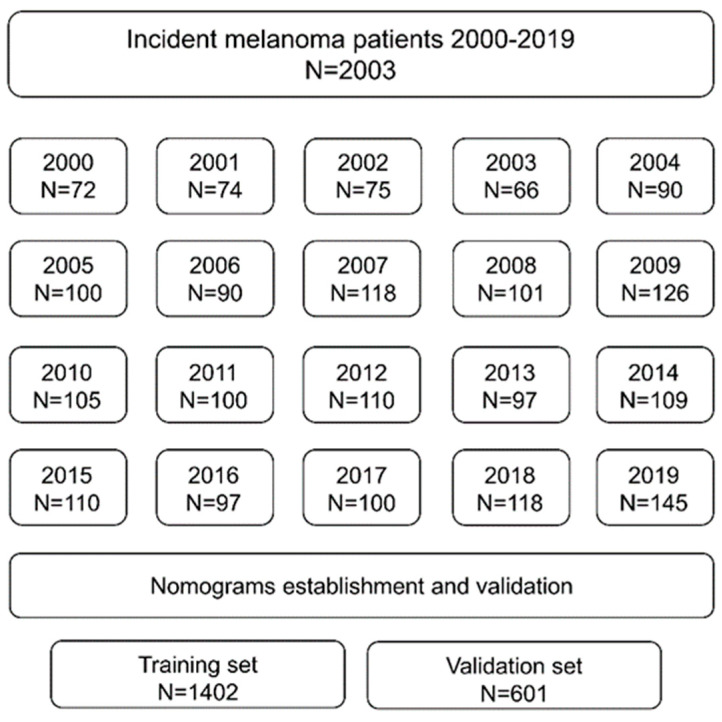
Flowchart showing the patients included in this study. N—number.

A total of 2003 patients diagnosed with malignant melanoma between 2000 and 2019 were included in the analysis (see Figure 1). In terms of primary tumor characteristics, the most frequent histological subtype was superficial spreading melanoma (SSM), and the most frequent location was the trunk. During follow-up, 57.3% of patients in our study population did not develop metastases. For the 1153 patients who were alive at the end of the observation period, the median follow-up time was 11.4 years from melanoma diagnosis. There were no significant differences between the training (N = 1402) and validation (N = 601) sets for any variables (Table 1).

### 3.2. Kaplan–Meier Survival Analysis

**Figure 2:**
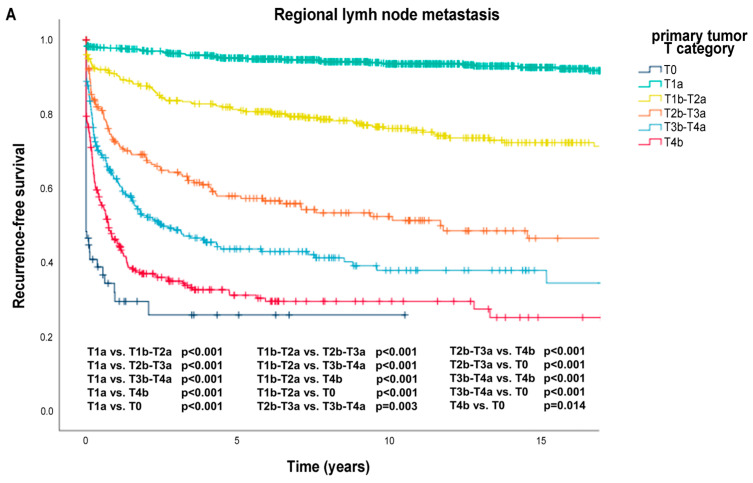

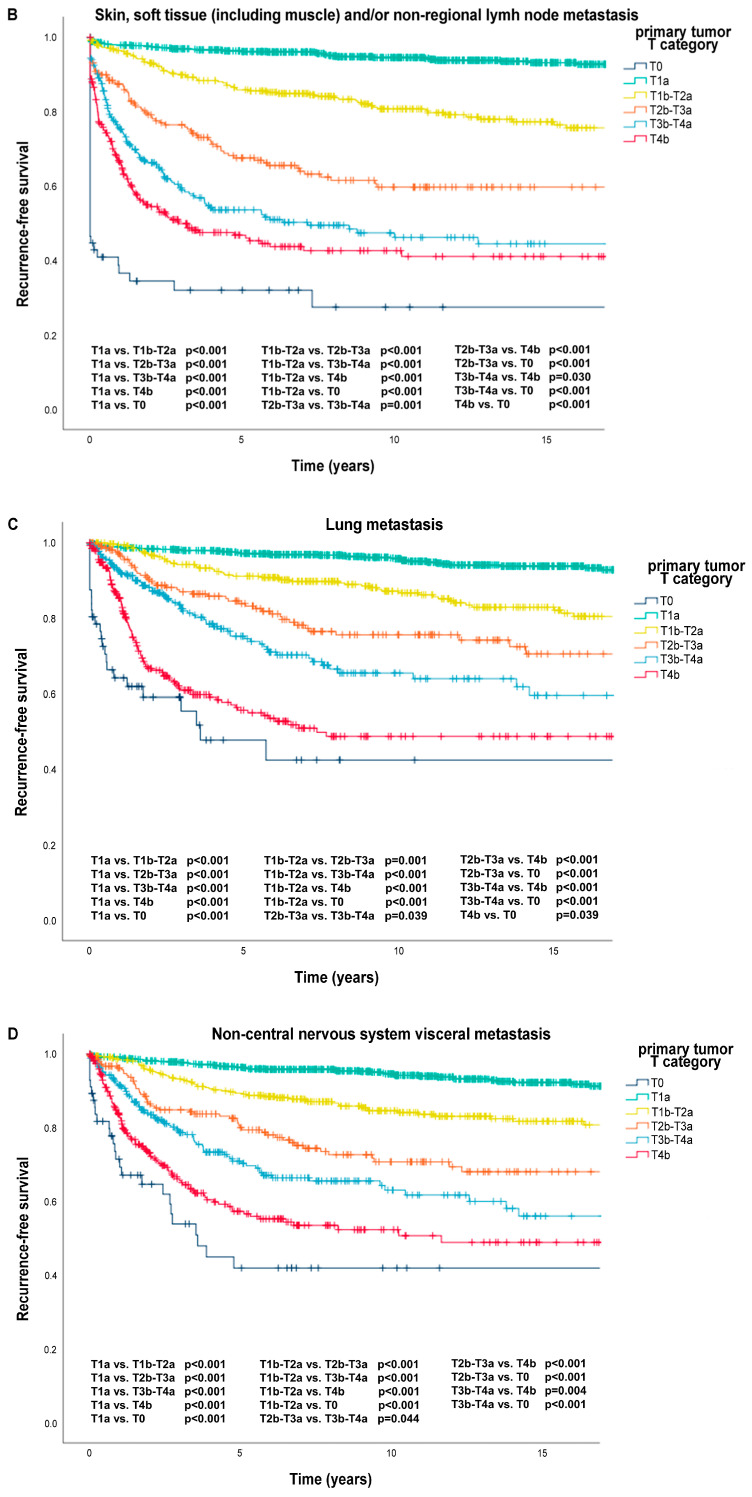

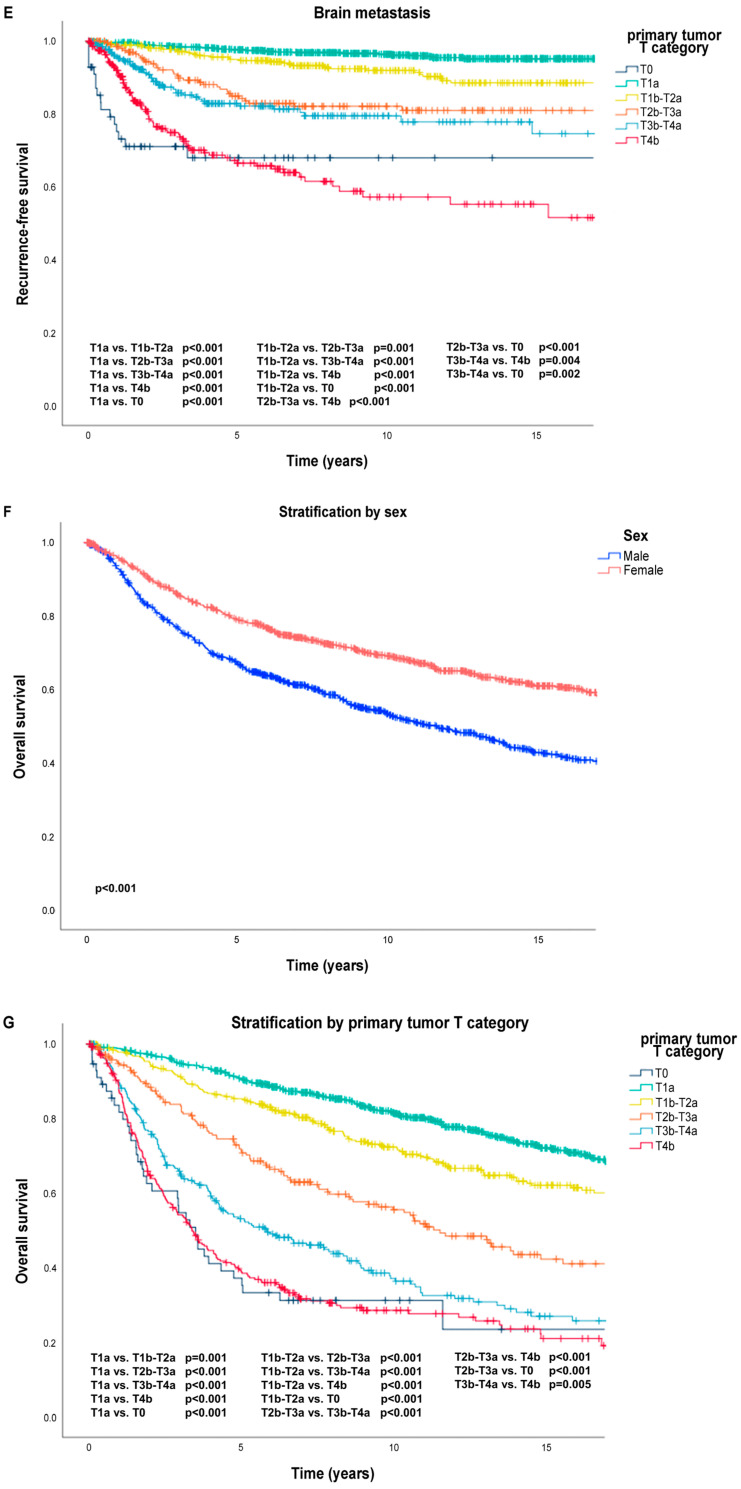

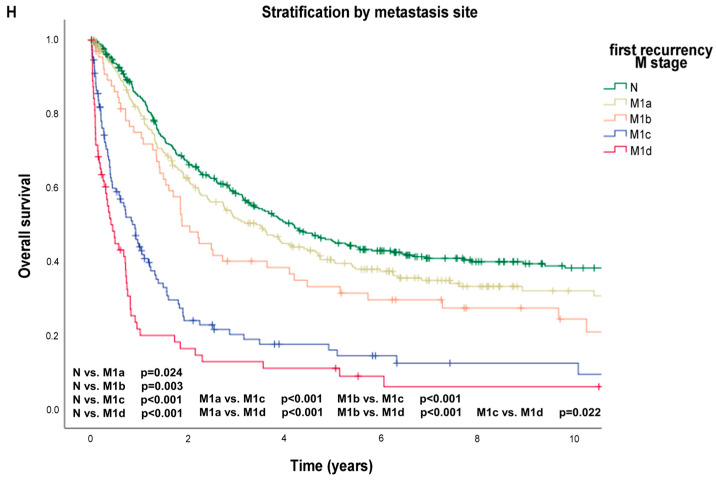
Recurrence-free survival is shown for regional lymph node metastasis (**A**); skin, soft tissue (including muscle) and/or non-regional lymph node metastasis (**B**); lung metastasis (**C**); non-central nervous system visceral metastasis (**D**); brain metastasis (**E**). Recurrence-free survival was stratified by the primary tumor’s American Joint Committee on Cancer (AJCC) T category. Overall survival is shown, stratified by patient sex (**F**) and primary tumor AJCC T category (**G**); the investigated period was from primary tumor to the end of the follow-up/death. Overall survival is shown stratified by metastasis site (**H**); the investigated period was from the first recurrence to the end of the follow-up/death.

Kaplan–Meier survival curves were plotted to demonstrate the differences in recurrence-free (RFS) and overall survival (OS) rates among the various risk groups (Figure 2A–E). The median RFS was 0.73 years [0.44; 1.03] for regional lymph node metastasis, 2.91 years [0.66; 5.15] for skin, soft tissue (including muscle) and/or non-regional lymph node metastasis, 7.34 years [5.13; 14.26] for lung metastasis, 11.64 years [8.05; 14.59] for non-central nervous system visceral metastasis and 14.36 years [12.65; 16.07] for brain metastasis. This analysis revealed a link between RFS and the AJCC 8th edition pT stage of the primary tumor for each recurrence type. Additionally, OS probabilities differed according to AJCC pT stage and patient sex (Figure 2F,G). The site of the first recurrence influenced the probability of OS from the time of recurrence (Figure 2H).

### 3.3. Cox Regression Analysis and Nomogram

Cox regression models were used to evaluate prognostic factors for melanoma recurrence at specific sites. The significant results of the univariate Cox regression analysis were incorporated into the multivariate model. During the multivariate regression analysis, we identified the significant independent factors that were then included in the nomogram.

#### 3.3.1. Cox Regression Analyses of Melanoma Recurrence in Regional Lymph Nodes

**Figure 3:**
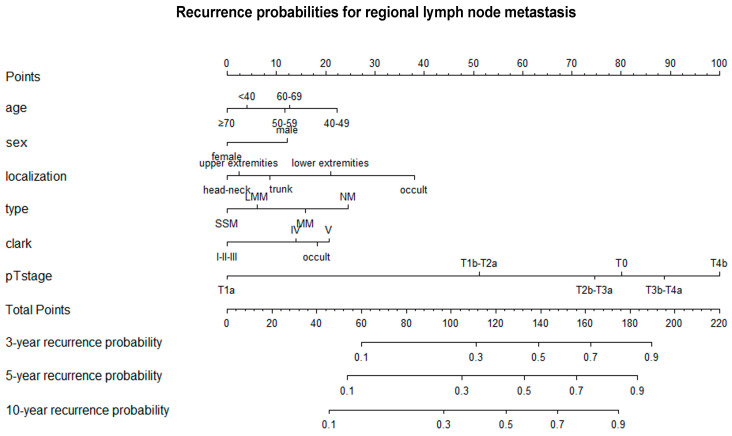
Nomogram for predicting three-, five-, and ten-year recurrence probabilities for regional lymph node metastasis. T—primary tumor.

Multivariate Cox regression analysis showed no significant difference in the occurrence of regional lymph node metastasis between melanoma cases diagnosed under the age of 40 and those diagnosed over the age of 50 (Table 2). However, the incidence of lymph node metastasis was significantly higher in cases diagnosed in the 40–49 age group than in cases diagnosed under the age of 40 (40–49 years vs. <40 years: hazard ratio (HR) 1.54 [1.11; 2.12]). Men were found to be at a higher risk of lymph node metastasis than women (male vs. female: HR 1.34 [1.13; 1.58]). Lymph node metastasis occurred with similar frequency in primary melanomas of the head and neck, upper extremities, and trunk, but more frequently in primary melanomas of the lower extremities or when the primary tumor site could not be identified (lower extremities vs. head and neck: HR 1.63 [1.23; 2.15], occult vs. head-neck: HR 4.86 [3.02; 6.72]). The incidence of lymph node metastasis was significantly lower in the SSM histological subtype than in the other subtypes, except for LMM (NM vs. SSM: HR 1.79 [1.38; 2.32]). Clark IV and V invasion levels were associated with a significantly higher probability of lymph node metastasis than Clark I-III invasion levels (Clark level V vs. I-III: HR 1.60 [1.20; 2.13]; Clark level IV vs. I-III: HR 1.39 [1.11; 1.74]). The probability of lymph node metastasis was closely correlated with the AJCC T category (T4b vs. T1a: HR 10.66 [7.13; 15.93]; T3b-T4a vs. T1a: HR 8.26 [5.57; 12.23]; T2b-T3a vs. T1a: HR 5.92 [4.02; 8.71]; T1b-T2a vs. T1a: HR 3.39 [2.39; 4.81]). Figure 3 shows a nomogram that integrates the independent variables to predict the probability of regional lymph node metastasis at 3, 5 and 10 years after melanoma diagnosis.

#### 3.3.2. Cox Regression Analyses of Melanoma Recurrence in the Skin, Soft Tissue (Including Muscle), and/or Non-Regional Lymph Nodes

**Figure 4:**
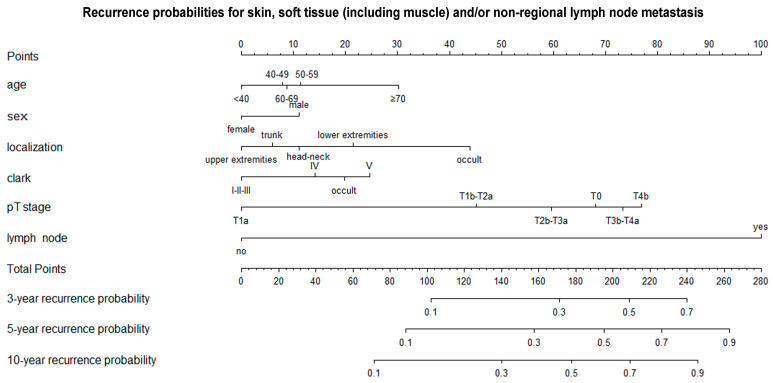
Nomogram for predicting three-, five-, and ten-year recurrence probabilities for skin, soft tissue (including muscle) and/or non-regional lymph node metastasis. T—primary tumor.

The likelihood of skin, soft tissue (including muscle) and/or non-regional lymph node metastases (equivalent to stage M1a in the 8th edition of the AJCC) was significantly higher in melanoma patients diagnosed at over 70 years of age than in those diagnosed at under 40 years of age (≥70 years vs. <40 years: HR 1.75 [1.23; 2.48]) (Table 3). For other age groups, there was no significant difference in the probability of this type of metastasis between melanoma diagnosed under the age of 40 and melanoma diagnosed at 40 years of age or older. Men were more likely to experience such metastases than women. These metastases occurred with similar frequency in primary melanomas of the head and neck region, extremities, and trunk, but more frequently in occult primary tumors. Histological subtype did not influence the incidence of this type of metastasis. Clark V invasion levels were associated with a significantly higher likelihood of these metastases than Clark I–III invasion levels. The odds of these metastases showed a strong statistical correlation with the AJCC T category. The probability of distant metastasis in the AJCC M1a category was significantly higher in cases of regional lymph node metastasis (yes vs. no: HR5.70 [4.48; 7.25]). Figure 4 shows a nomogram for predicting the probability of recurrence within three, five and ten years after melanoma diagnosis at sites classified as AJCC M1a stage.

#### 3.3.3. Cox Regression Analyses of Melanoma Recurrence in the Lung, Non-Central Nervous System Visceral Sites and the Brain

**Figure 5:**
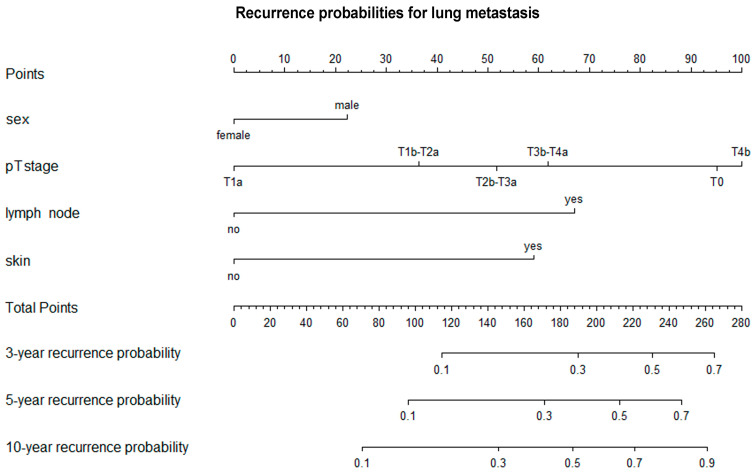
Nomogram for predicting three-, five-, and ten-year recurrence probabilities for lung metastasis. T—primary tumor.

**Figure 6:**
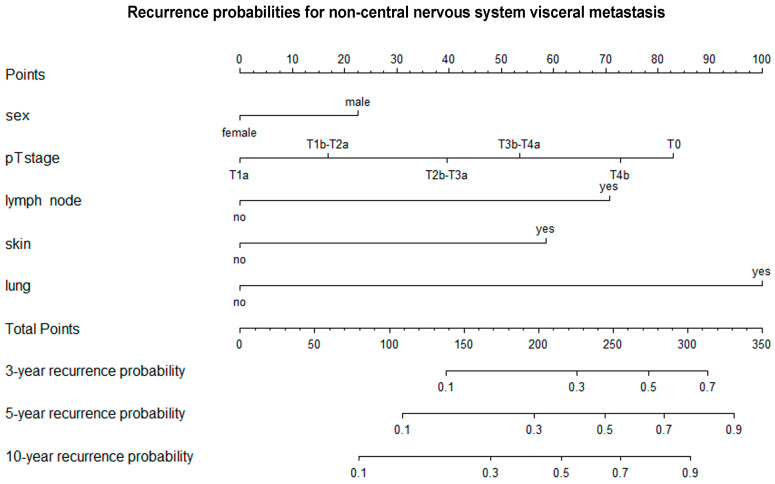
A nomogram that predicts the three-, five-, and ten-year recurrence probabilities for non-central nervous system visceral metastases. T—primary tumor.

**Figure 7:**
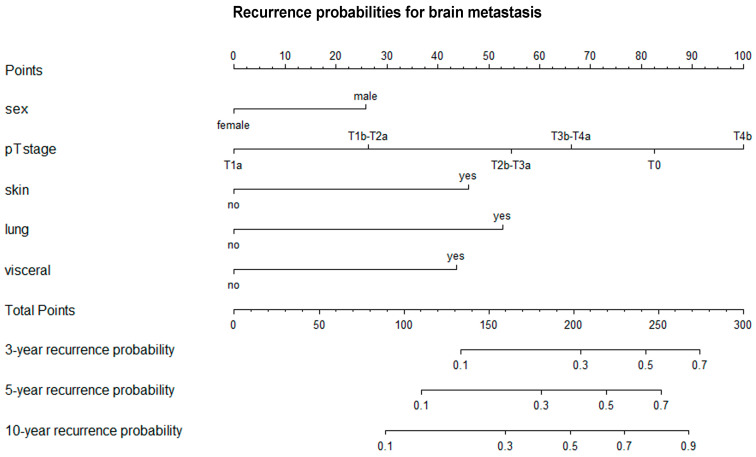
Nomogram for predicting three-, five-, and ten-year recurrence probabilities for brain metastasis. T—primary tumor.

Using multivariate Cox regression models, we found that the likelihood of lung, visceral or brain metastasis did not differ according to age at primary melanoma diagnosis, primary tumor location, histological subtype or Clark invasion level (Table 4, Table 5 and Table 6). However, the probability of all types of distant metastasis showed a strong statistical correlation with the AJCC T category (lung metastasis: T4b vs. T1a: HR 5.53 [3.29; 9.30]; T3b-T4a vs. T1a: HR 2.93 [1.73; 4.94]; T2b-T3a vs. T1a: HR 2.49 [1.50; 4.13]; T1b-T2a vs. T1a: HR 3.39 1.94 [1.24; 3.02]; T0 vs. T1a: HR 2.32 [1.03; 4.00]; visceral metastasis: T4b vs. T1a: HR 2.46 [1.51; 4.00]; T3b-T4a vs. T1a: HR 1.93 [1.19; 3.12]; T2b-T3a vs. T1a: HR 1.69 [1.05; 2.69]; T0 vs. T1a: HR 2.86 [1.44; 5.66]; brain metastasis: T4b vs. T1a: HR 5.00 [2.73; 9.18]; T3b-T4a vs. T1a: HR 2.82 [1.54; 5.19]; T2b-T3a vs. T1a: HR 2.32 [1.28; 4.20]; T0 vs. T1a: HR 3.29 [1.33; 8.13]) and was significantly higher in men than in women (lung metastasis: male vs. female: HR 1.30 [1.03; 1.63]; visceral metastasis: male vs. female: HR 1.29 [1.04; 1.61]; brain metastasis: male vs. female: HR 1.49 [1.13; 1.96]). The likelihood of lung metastasis was notably higher in cases involving regional lymph node metastasis (yes vs. no: HR 2.95 [2.18; 4.00]), skin and soft tissue (including muscle) and/or non-regional lymph node metastasis (yes vs. no: HR 2.63 [2.02; 3.43]). Visceral metastasis was significantly more likely in cases of regional lymph node metastasis (yes vs. no: HR 2.62 [1.96; 3.51]), skin and soft tissue (including muscle) and/or non-regional lymph node metastasis (yes vs. no: HR 2.31 [1.79; 2.79]) and lung metastasis (yes vs. no: HR 4.00 [3.18; 5.04]). Likewise, the likelihood of brain metastasis was higher in cases involving skin, soft tissue (including muscle) and/or non-regional lymph node metastases (yes vs. no: HR 2.11 [1.53; 2.92]), lung metastases (yes vs. no: HR 2.45 [1.79; 3.36]) and visceral metastases (yes vs. no: HR 2.10 [1.53; 2.89]). Nomograms were created to combine the independent variables and predict the likelihood of lung, visceral and brain metastases occurring within three, five and ten years (Figure 5, Figure 6 and Figure 7).

### 3.4. Nomogram Validation

The nomograms were evaluated and validated using multiple metrics. First of all, the C-index of the training cohort and validation cohort in case of regional lymph node metastasis nomogram was 0.81 [95% CI 0.78; 0.84] and 0.83 [95% CI 0.79; 0.87], respectively. In the case of distant skin, soft tissue (including muscle) and/or non-regional lymph node metastases, the C-index was 0.84 [0.81; 0.87] in the training cohort and 0.84 [0.80; 0.88] in the validation cohort, while in the case of lung metastasis, it was 0.84 [0.81; 0.87] and 0.83 [0.79; 0.88], for visceral metastasis 0.86 [0.84; 0.90] and 0.85 [0.81; 0.90], and for brain metastasis 0.85 [0.82; 0.89] and 0.82 [0.76; 0.89], respectively.

In the training cohort, the AUC of predicted nomogram for 3-, 5- and 10-year recurrence probability of lymph node metastasis were 0.864 [0.846; 0.882], 0.878 [0.861; 0.895], and 0.887 [0.870; 0.904], while in the validation cohort, the AUC for 3-, 5- and 10-year recurrence probability were 0.881 [0.847; 0.915], 0.877 [0.843; 0.911] and 0.891 [0.858; 0.924] (Figure S1A,B). In case of distant skin, soft tissue (including muscle) and/or non-regional lymph node metastases nomogram the AUC for 3-, 5- and 10-year recurrence probability were 0.898 [0.876; 0.920], 0.921 [0.901; 0.941] and 0.934 [0.916; 0.952] in the training set, and 0.883 [0.846; 0.920], 0.888 [0.852; 0.924] and 0.900 [0.865; 0.935] in the validation set (Figure S1C,D). In case of lung metastases nomogram, the AUC for 3-, 5- and 10-year recurrence probability were 0.884 [0.855; 0.913], 0.890 [0.862; 0.918] and 0.931 [0.908; 0.954] in the training set, and 0.883 [0.840; 0.926], 0.870 [0.826; 0.914] and 0.883 [0.840; 0.926] in the validation set (Figure S1E,F). In case of visceral metastases nomogram, the AUC for 3-, 5- and 10-year recurrence probability were 0.901 [0.876; 0.926], 0.910 [0.886 0.934] and 0.942 [0.923; 0.961] in the training set, and 0.862 [0.817; 0.907], 0.879 [0.836; 0.922] and 0.917 [0.881; 0.953] in the validation set (Figure S1G,H). In the case of brain metastases nomogram the AUC for 3-, 5- and 10-year recurrence probability were 0.881 [0.847; 0.915], 0.899 [0.867; 0.931] and 0.939 [0.914; 0.964] in the training set, and 0.855 [0.798; 0.912], 0.868 [0.813; 0.923] and 0.885 [0.798; 0.912] in the validation set (Figure S1I,J). The C-index and ROC curves indicated that the models had good discriminatory power.

Calibration plots, which were used to predict the probability of recurrence in the training and validation cohorts, showed good agreement for all types of melanoma recurrence (Figure S2A,J). DCA demonstrated the significant net benefit of the predictive model for all nomogram types in both cohorts (Figure S3A,J).

## 4. Discussion

Cutaneous melanoma can be characterized by an increasing incidence associated with an aging population and high rate of UV exposure, a high cure rate when diagnosed and completely excised at an early stage, and a poor prognosis in advanced cases [1,2,3]. Patterns concerning the probability, timing and site of disease recurrence allow us to categorize patients according to their risk of relapse and their need for follow-up care and treatment [4,5,6,20]. The most powerful prognostic variables are represented in the American Joint Committee on Cancer (AJCC) TNM classification (8th edition) [21]. However, existing prognostic heterogeneity supports continued research for independent, readily available prognostic factors that could improve clinical decision-making. Identifying prognostic factors that are independent of the AJCC T, N and M categories and developing risk prediction models are highly relevant to improving morbidity and mortality outcomes in melanoma patients [22,23]. Furthermore, identifying significant prognostic factors across different databases (geographical regions) can inform molecular research. Recently, several risk prediction models have been published that demonstrate good predictive performance for survival in patients with non-metastatic [11,24] or metastatic [12,25,26,27] melanoma; elderly patients [28,29]; and patients with specified histopathological types [30,31,32] or localizations [10,15,33,34,35,36,37] of melanoma. In most studies, the Surveillance, Epidemiology, and End Results (SEER) database was used to obtain patient demographic and clinical information. The most common independent prognostic factors were age, AJCC stage, primary site, sex, histological subtype and marital status. Selected clinical outcomes included overall survival, cancer-specific survival and, in some studies, recurrence-free survival [9,11,38] and early death [12,27,28]. Notably, the published nomogram models showed that Breslow thickness, ulceration of the primary tumor, histological subtype and primary site were independent predictors of survival in patients with metastatic melanoma [25,26,27]. Our previous studies also found that primary tumor characteristics maintained their prognostic power as independent variables for survival in metastatic melanoma patients treated with immune checkpoint inhibitors or BRAF and MEK inhibitor combinations [39,40].

For the present study, we extracted demographic, clinicopathological and outcome data from a university hospital-based melanoma registry in order to identify independent prognostic factors that are readily available and associated with disease recurrence. We employed univariate and multivariate Cox regression models and then constructed a nomogram model to predict selected patient outcomes. This study is novel in that we aimed to identify independent prognostic factors associated with melanoma recurrence at specific sites, and to construct nomograms to predict recurrence-free survival. We believe that these nomograms could be useful in providing a more personalized prognosis assessment and in supporting clinical decisions regarding adjuvant treatment and follow-up.

We found that the risk of melanoma recurrence in regional lymph nodes can be more accurately assessed by considering additional clinical parameters alongside the thickness and ulceration of the primary tumor. Furthermore, our results suggest that the site and histological subtype of primary melanomas, as well as the Clark invasion level, provide valuable insights into melanoma biology that remain largely unexplored. Although risk prediction models developed to predict sentinel lymph node status using clinicopathological features have been shown to reduce unnecessary sentinel lymph node biopsies (SLNB), there is concern that clinical nomograms may not be accurate enough to provide clear benefits to patients with early-stage melanoma [7,41,42]. The clinicopathological and gene expression profile (CP-GEP) model may be more effective in identifying low-risk melanoma patients who can safely avoid SLNB [8]. This model incorporates two clinicopathological factors: age and Breslow thickness. Notably, the CP-GEP model considers an age of less than 40 years to be an indication for SLNB. However, our study showed that the 40–49 age group was at a higher risk of regional lymph node metastasis than those diagnosed under 40 years of age (although occult and clinically detected lymph node metastasis cases were not considered separately). It is also worth mentioning that integrating artificial intelligence platforms into risk prediction models has recently gained attention in predicting SLNB positivity [43].

As expected, we found that patients’ overall survival estimates depended on the site of metastases [44,45]. Metastasis in regional lymph nodes increased the risk of metastasis at a distant site, which in turn increased the likelihood of metastasis developing in additional sites. It should be noted that the outcomes of patients in this study reflect the era before adjuvant treatment with immune checkpoint inhibitors and BRAF and MEK inhibitor combinations. As previously mentioned, there is evidence that primary tumor characteristics are independent prognostic factors for overall survival in patients with advanced melanoma [25,26,27,39,40]. In this study, we found that the AJCC 8th edition T classification was an independent predictor of the risk of melanoma recurrence at distant sites. Furthermore, while the other features of the primary tumor did not predict the risk of distant metastases, except for the prognostic significance of Clark invasion level and the occult nature of primary melanoma in the occurrence of distant skin, soft tissue (including muscle) and/or non-regional lymph node metastases, we should bear in mind that these characteristics had a prognostic effect on the risk of regional lymph node metastases. This was also an independent predictor of lung and non-central nervous system visceral metastases.

In terms of age, patients over 70 were found to have a relatively lower risk of regional lymph node metastasis than those under 40. However, they exhibited a significantly higher risk of distant skin, soft tissue (including muscle) and/or non-regional lymph node metastases. Interestingly, age-related alterations in lymphatic integrity may explain this finding [46]. Finally, we found that patient sex was an independent prognostic factor for melanoma recurrence in all cases. The prognostic value of sex for survival in melanoma patients has been evaluated in multiple studies, with some reporting it to be an independent prognostic variable and others not [47,48]. However, a recent study employing a machine learning model found that incorporating patient age and sex into the TNM prognostic system was more accurate at predicting cancer-specific survival in patients with cutaneous melanoma than TNM staging alone [22]. A worse prognosis for male patients than female patients with melanoma may be due to differences in hormonal status, as well as sex-related genetic and epigenetic factors that can influence tumor growth and the anti-tumor immune response [49].

This study’s strength lies in its use of multivariate logistic regression analysis to identify independent prognostic factors for disease recurrence in melanoma patients at specified sites. Nomograms were constructed and validated by integrating these variables. A limitation of our study is that it was retrospective and single-center; further prospective validation on independent melanoma cohorts is required. As the definitions of mitotic index and microscopic satellites varied in the histological reports of primary tumors, these prognostic factors could not be included in the nomogram. We were unable to distinguish between occult and clinically detected lymph node metastases because SLNB was not consistently performed on all patients with melanoma measuring 1 mm or above in thickness. Data on interferon therapy, radiotherapy and chemotherapy were excluded from the statistical analysis due to heterogeneity in the indications and therapeutic regimens applied. Furthermore, melanoma-specific survival could not be evaluated as an outcome measure.

## 5. Conclusions

Stratifying cutaneous melanoma patients according to prognostic biomarkers could improve disease outcomes. Our study suggests that risk prediction models can be successfully constructed using clinicopathological variables, potentially increasing the accuracy of prognostic considerations. However, the created nomograms require further validation before they can be used in daily clinical practice. Moreover, there is an urgent need to identify molecular biomarkers that can be easily measured using standardized assays and incorporated into a multivariate prognostic model.

## Figures and Tables

**Table 1:** Characteristics of the study population.

Variables, N (%)	Categories	Patients N = 2003	Training Set N = 1402	Validation Set N = 601	*p*-Values
Age (years)	<40	265 (13.2)	172 (12.3)	93 (15.5)	0.193
	40–49	291 (14.5)	196 (14.0)	95 (15.8)	
	50–59	388 (19.4)	273 (19.5)	115 (19.1)	
	60–69	476 (23.8)	344 (24.5)	132 (22.0)	
	≥70	583 (29.1)	417 (29.7)	166 (27.6)	
Sex	male	941 (47.0)	649 (46.3)	292 (48.6)	0.346
	female	1062 (53.0)	753 (53.7)	309 (51.4)	
Localization of primary tumor	head and neck	338 (16.9)	241 (17.2)	97 (16.1)	0.167
	upper extremities	324 (16.2)	243 (17.3)	81 (13.5)	
	lower extremities	435 (21.7)	303 (21.6)	132 (22.0)	
	trunk	850 (42.4)	579 (41.3)	271 (45.1)	
	occult	56 (2.8)	36 (2.6)	20 (3.3)	
Histological subtype	SSM	1105 (55.2)	772 (55.1)	333 (55.4)	0.525
	LMM	133 (6.6)	99 (7.1)	34 (5.7)	
	NM	292 (14.6)	197 (14.1)	95 (15.8)	
	MM	473 (23.6)	334 (23.8)	139 (23.1)	
Clark level	I-II-III	1260 (62.9)	869 (62.0)	391 (65.0)	0.149
	IV	394 (19.7)	274 (19.5)	120 (20.0)	
	V	160 (8.0)	122 (8.7)	38 (6.3)	
	occult	56 (2.8)	36 (2.6)	20 (3.3)	
	unknown	133 (6.6)	101 (7.2)	32 (5.3)	
AJCC 8th edition T category	T1a	768 (38.3)	540 (38.5)	228 (37.9)	0.239
	T1b-T2a	375 (18.7)	268 (19.1)	107 (17.8)	
	T2b-T3a	219 (10.9)	142 (10.1)	77 (12.8)	
	T3b-T4a	267 (13.3)	190 (13.6)	77 (12.8)	
	T4b	276 (13.8)	205 (14.6)	71 (11.8)	
	T0	56 (2.8)	36 (2.6)	20 (3.3)	
	unknown	42 (2.1)	21 (1.5)	21 (3.5)	
Metastasis during follow-up	no	1147 (57.3)	801 (57.1)	346 (57.6)	0.856
	yes	856 (42.7)	601 (42.9)	255 (42.4)	
Death during follow-up	no	1153 (57.6)	803 (57.3)	350 (58.2)	0.690
	yes	850 (42.4)	599 (42.7)	251 (41.8)	

N—number; SSM—superficial spreading melanoma; LMM—lentigo maligna melanoma; NM-nodular melanoma; MM—unclassified malignant melanoma or histological subtype other than SSM, LMM or NM or no evidence of primary tumor; AJCC—American Joint Committee on Cancer.

**Table 2:** Univariate and multivariate Cox regression analyses of melanoma recurrence in regional lymph nodes.

Variables	Categories	Univariate		Multivariate	
		HR [95% CI]	*p*-Value	HR [95% CI]	*p*-Value
Age (years)	40–49/<40	**1.64 [1.19; 2.26]**	**0.002**	**1.54 [1.11; 2.12]**	**0.009**
	50–59/<40	**1.47 [1.08; 2.01]**	**0.015**	1.20 [0.88; 1.64]	0.249
	60–69/<40	**1.91 [1.43; 2.57]**	**<0.001**	1.24 [0.92; 1.67]	0.158
	≥70/<40	**1.56 [1.16; 2.10]**	**0.003**	0.92 [0.68; 1.25]	0.589
Sex	male/female	**1.53 [1.31; 1.79]**	**<0.001**	**1.34 [1.13; 1.58]**	**0.001**
Localization of primary tumor	upper extremities/head-neck	1.11 [0.81; 1.52]	0.499	1.05 [0.76; 1.45]	0.769
	lower extremities/head-neck	**1.91 [1.46; 2.50]**	**<0.001**	**1.63 [1.23; 2.15]**	**<0.001**
	trunk/head-neck	**1.37 [1.06; 1.77]**	**0.016**	1.22 [0.93; 1.61]	0.148
	occult/head-neck	**6.94 [4.70; 10.26]**	**<0.001**	**4.86 [3.02; 6.72]**	**<0.001**
Histological subtype	LMM/SSM	0.56 [0.31; 1.00]	0.051	1.15 [0.62; 2.15]	0.661
	NM/SSM	**4.93 [3.94; 6.17]**	**<0.001**	**1.79 [1.38; 2.32]**	**<0.001**
	MM/SSM	**5.38 [4.44; 6.53]**	**<0.001**	**1.47 [1.15; 1.87]**	**<0.001**
Clark level	IV/I-II-III	**4.31 [3.55; 5.23]**	**<0.001**	**1.39 [1.11; 1.74]**	**0.004**
	V/I-II-III	**7.01 [5.51; 8.92]**	**<0.001**	**1.60 [1.20; 2.13]**	**0.001**
	occult/I-II-III	**11.22 [7.92; 15.88]**	**<0.001**	**1.54 [1.13; 2.10]**	**0.007**
AJCC 8th edition T category	T1b-T2a/T1a	**3.97 [2.83; 5.58]**	**<0.001**	**3.39 [2.39; 4.81]**	**<0.001**
	T2b-T3a/T1a	**9.39 [6.70; 13.17]**	**<0.001**	**5.92 [4.02; 8.71]**	**<0.001**
	T3b-T4a/T1a	**13.88 [10.08; 19.11]**	**<0.001**	**8.26 [5.57; 12.23]**	**<0.001**
	T4b/T1a	**20.67 [15.11; 28.27]**	**<0.001**	**10.66 [7.13; 15.93]**	**<0.001**
	T0/T1a	**13.22 [8.09; 21.62]**	**<0.001**	**6.70 [3.82; 11.74]**	**<0.001**

Significant results are in bold. HR [95% CI]—hazard ratio with 95% confidence intervals; LMM—lentigo maligna melanoma; SSM—superficial spreading melanoma; NM—nodular melanoma; MM—unclassified malignant melanoma or no evidence of primary tumor; AJCC—American Joint Committee on Cancer; T—primary tumor.

**Table 3:** Univariate and multivariate Cox regression analyses of melanoma recurrence at sites classified as AJCC M1a stage.

Variables	Categories	Univariate		Multivariate	
		HR [95% CI]	*p*-Value	HR [95% CI]	*p*-Value
Age (years)	40–49/<40	**1.56 [1.06; 2.28]**	**0.023**	1.18 [0.80; 1.73]	0.408
	50–59/<40	**1.67 [1.17; 2.40]**	**0.005**	1.26 [0.88; 1.81]	0.213
	60–69/<40	**2.03 [1.44; 2.86]**	**<0.001**	1.20 [0.84; 1.71]	0.315
	≥70/<40	**2.27 [1.62; 3.18]**	**<0.001**	**1.75 [1.23; 2.48]**	**0.002**
Sex	male/female	**1.46 [1.23; 1.74]**	**<0.001**	**1.21 [1.01; 1.47]**	**0.049**
Localization of primary tumor	upper extremities/head-neck	0.80 [0.56; 1.14]	0.216	0.80 [0.55; 1.16]	0.242
	lower extremities/head-neck	**1.74 [1.31; 2.32]**	**<0.001**	1.19 [0.88; 1.61]	0.249
	trunk/head-neck	1.06 [0.80; 1.39]	0.700	0.90 [0.67; 1.21]	0.488
	occult/head-neck	**7.01 [4.70; 10.45]**	**<0.001**	**8.21 [4.77; 14.14]**	**<0.001**
Histological subtype	LMM/SSM	0.58 [0.30; 1.10]	0.093	0.99 [0.49; 1.97]	0.969
	NM/SSM	**4.64 [3.61; 5.94]**	**<0.001**	1.30 [0.98; 1.73]	0.066
	MM/SSM	**4.70 [3.79; 5.83]**	**<0.001**	1.19 [0.91; 1.54]	0.201
Clark level	IV/I-II-III	**4.43 [3.56; 5.51]**	**<0.001**	1.25 [0.97; 1.61]	0.091
	V/I-II-III	**7.30 [5.57; 9.56]**	**<0.001**	**1.49 [1.08; 2.06]**	**0.017**
	occult/I-II-III	**13.48 [9.43; 19.28]**	**<0.001**	1.35 [0.94; 1.93]	0.102
AJCC 8th edition T category	T1b-T2a/T1a	**3.55 [2.44; 5.18]**	**<0.001**	**2.17 [1.45; 3.24]**	**<0.001**
	T2b-T3a/T1a	**7.89 [5.43; 11.46]**	**<0.001**	**2.67 [1.71; 4.16]**	**<0.001**
	T3b-T4a/T1a	**12.61 [8.91; 17.85]**	**<0.001**	**3.31 [2.11; 5.18]**	**<0.001**
	T4b/T1a	**16.55 [11.75; 23.30]**	**<0.001**	**3.48 [2.19; 5.53]**	**<0.001**
	T0/T1a	**11.33 [6.54; 19.61]**	**<0.001**	**2.98 [1.56; 5.70]**	**0.001**
Regional lymph node metastasis	yes/no	**11.64 [9.44; 14.36]**	**<0.001**	**5.70 [4.48; 7.25]**	**<0.001**

Significant results are in bold. HR [95% CI]—hazard ratio with 95% confidence intervals; LMM—lentigo maligna melanoma; SSM—superficial spreading melanoma; NM—nodular melanoma; MM—unclassified malignant melanoma or no evidence of primary tumor; AJCC—American Joint Committee on Cancer; T—primary tumor; M1a—metastases to skin, soft tissue (including muscle), and/or non-regional lymph nodes.

**Table 4:** Univariate and multivariate Cox regression analyses of melanoma recurrence in the lung.

Variables	Categories	Univariate		Multivariate	
		HR [95% CI]	*p*-Value	HR [95% CI]	*p*-Value
Age (years)	40–49/<40	**1.77 [1.14; 2.77]**	**0.012**	1.23 [0.78; 1.93]	0.373
	50–59/<40	**1.75 [1.14; 2.68]**	**0.010**	1.32 [0.85; 2.04]	0.214
	60–69/<40	**2.17 [1.44; 3.27]**	**<0.001**	1.39 [0.91; 2.11]	0.125
	≥70/<40	**2.06 [1.36; 3.12]**	**0.001**	1.35 [0.88; 2.07]	0.163
Sex	male/female	**1.82 [1.47; 2.25]**	**<0.001**	**1.30 [1.03; 1.63]**	**0.028**
Localization of primary tumor	upper extremities/head-neck	0.73 [0.48; 1.09]	0.124	0.77 [0.50; 1.17]	0.214
	lower extremities/head-neck	0.99 [0.69; 1.40]	0.993	1.11 [0.81; 1.54]	0.120
	trunk/head-neck	1.07 [0.78; 1.46]	0.675	0.89 [0.63; 1.25]	0.483
	occult/head-neck	**5.91 [3.67; 9.53]**	**<0.001**	2.61 [0.95; 2.87]	0.061
Histological subtype	LMM/SSM	0.85 [0.46; 1.58]	0.613	1.11 [0.66; 1.61]	0.440
	NM/SSM	**3.91 [2.90; 5.27]**	**<0.001**	1.17 [0.69; 1.36]	0.864
	MM/SSM	**3.83 [2.96; 4.95]**	**<0.001**	1.20 [0.60; 1.31]	0.221
Clark level	IV/I-II-III	**3.60 [2.77; 4.67]**	**<0.001**	1.17 [0.86; 1.59]	0.331
	V/I-II-III	**5.94 [4.29; 8.24]**	**<0.001**	1.25 [0.84; 1.85]	0.269
	occult/I-II-III	**11.71 [7.59; 18.07]**	**<0.001**	1.28 [0.85; 1.94]	0.243
AJCC 8th edition T category	T1b-T2a/T1a	**2.85 [1.89; 4.30]**	**<0.001**	**1.94 [1.24; 3.02]**	**0.004**
	T2b-T3a/T1a	**5.25 [3.43; 8.04]**	**<0.001**	**2.49 [1.50; 4.13]**	**<0.001**
	T3b-T4a/T1a	**7.78 [5.24; 11.53]**	**<0.001**	**2.93 [1.73; 4.94]**	**<0.001**
	T4b/T1a	**15.24 [10.55; 22.01]**	**<0.001**	**5.53 [3.29; 9.30]**	**<0.001**
	T0/T1a	**6.27 [3.04; 12.93]**	**<0.001**	**2.32 [1.03; 4.00]**	**0.043**
Regional lymph node metastasis	yes/no	**8.98 [7.07; 11.41]**	**<0.001**	**2.95 [2.18; 4.00]**	**<0.001**
Distant metastasis at sites M1a	yes/no	**7.66 [6.13; 9.57]**	**<0.001**	**2.63 [2.02; 3.43]**	**<0.001**

Significant results are in bold. HR [95% CI]—hazard ratio with 95% confidence intervals; LMM—lentigo maligna melanoma; SSM—superficial spreading melanoma; NM—nodular melanoma; MM—unclassified malignant melanoma or no evidence of primary tumor; AJCC—American Joint Committee on Cancer; T—primary tumor; M1a—metastases to skin, soft tissue (including muscle), and/or non-regional lymph nodes.

**Table 5:** Univariate and multivariate Cox regression analyses of melanoma recurrence at non-central nervous system visceral sites.

Variables	Categories	Univariate		Multivariate	
		HR [95% CI]	*p*-Value	HR [95% CI]	*p*-Value
Age (years)	40–49/<40	**1.49 [1.01; 2.02]**	**0.048**	1.04 [0.69; 1.55]	0.864
	50–59/<40	1.36 [0.93; 1.99]	0.112	0.99 [0.67; 1.46]	0.953
	60–69/<40	**1.77 [1.23; 2.54]**	**0.002**	1.13 [0.78; 1.65]	0.514
	≥70/<40	**1.61 [1.12; 2.33]**	**0.010**	1.09 [0.75; 1.60]	0.646
Sex	male/female	**1.83 [1.50; 2.24]**	**<0.001**	**1.29 [1.04; 1.61]**	**0.021**
Localization of primary tumor	upper extremities/head-neck	0.88 [0.60; 1.29]	0.502	0.84 [0.59; 1.20]	0.338
	lower extremities/head-neck	1.08 [0.77; 1.52]	0.657	1.08 [0.73; 1.61]	0.707
	trunk/head-neck	1.16 [0.86; 1.57]	0.337	1.10 [0.79; 1.53]	0.583
	occult/head-neck	**5.00 [3.13; 7.98]**	**<0.001**	3.35 [0.97; 4.05]	0.059
Histological subtype	LMM/SSM	0.86 [0.48; 1.56]	0.063	1.18 [0.62; 2.27]	0.613
	NM/SSM	**4.11 [3.10; 5.45]**	**<0.001**	1.10 [0.80; 1.53]	0.556
	MM/SSM	**4.04 [3.17; 5.15]**	**<0.001**	1.19 [0.88; 1.62]	0.250
Clark level	IV/I-II-III	**3.13 [2.44; 4.01]**	**<0.001**	1.02 [0.73; 1.30]	0.862
	V/I-II-III	**5.51 [4.06; 7.46]**	**<0.001**	1.18 [0.82; 1.69]	0.384
	occult/I-II-III	**8.49 [5.58; 12.92]**	**<0.001**	1.04 [0.70; 1.54]	0.853
AJCC 8th edition T category	T1b-T2a/T1a	**2.66 [1.82; 3.88]**	**<0.001**	1.28 [0.84; 1.95]	0.259
	T2b-T3a/T1a	**5.07 [3.44; 7.47]**	**<0.001**	**1.69 [1.05; 2.69]**	**0.029**
	T3b-T4a/T1a	**7.24 [5.06; 10.36]**	**<0.001**	**1.93 [1.19; 3.12]**	**0.007**
	T4b/T1a	**11.40 [8.09; 16.08]**	**<0.001**	**2.46 [1.51; 4.00]**	**<0.001**
	T0/T1a	**8.56 [4.80; 15.27]**	**<0.001**	**2.86 [1.44; 5.66]**	**0.003**
Regional lymph node metastasis	yes/no	**10.04 [7.96; 12.67]**	**<0.001**	**2.62 [1.96; 3.51]**	**<0.001**
Distant metastasis at sites M1a	yes/no	**8.43 [6.81; 10.43]**	**<0.001**	**2.31 [1.79; 2.97]**	**<0.001**
Lung metastasis	yes/no	**10.81 [8.78; 13.30]**	**<0.001**	**4.00 [3.18; 5.04]**	**<0.001**

Significant results are in bold. HR [95% CI]—hazard ratio with 95% confidence intervals; LMM—lentigo maligna melanoma; SSM—superficial spreading melanoma; NM—nodular melanoma; MM—unclassified malignant melanoma or no evidence of primary tumor; AJCC—American Joint Committee on Cancer; T—primary tumor; M1a—metastases to skin, soft tissue (including muscle), and/or non-regional lymph nodes.

**Table 6:** Univariate and multivariate Cox regression analyses of melanoma recurrence in the brain.

Variables	Categories	Univariate		Multivariate	
		HR [95% CI]	*p*-Value	HR [95% CI]	*p*-Value
Age (years)	40–49/<40	**1.70 [1.01; 2.87]**	**0.046**	1.40 [0.82; 2.37]	0.218
	50–59/<40	**1.70 [1.03; 2.81]**	**0.037**	1.34 [0.80; 2.22]	0.265
	60–69/<40	**1.82 [1.12; 2.95]**	**0.017**	1.28 [0.77; 2.10]	0.341
	≥70/<40	**1.92 [1.19; 3.12]**	**0.008**	1.43 [0.87; 2.35]	0.159
Sex	male/female	**2.05 [1.58; 2.65]**	**<0.001**	**1.49 [1.13; 1.96]**	**0.005**
Localization of primary tumor	upper extremities/head-neck	0.87 [0.54; 1.42]	0.582	0.87 [0.53; 1.43]	0.583
	lower extremities/head-neck	1.11 [0.72; 1.71]	0.637	0.86 [0.55; 1.35]	0.520
	trunk/head-neck	1.16 [0.79; 1.71]	0.443	0.98 [0.64; 1.48]	0.906
	occult/head-neck	**4.54 [2.51; 8.24]**	**<0.001**	2.05 [0.98; 3.58]	0.058
Histological subtype	LMM/SSM	0.58 [0.23; 1.43]	0.235	0.79 [0.30; 2.08]	0.635
	NM/SSM	**3.81 [2.65; 5.49]**	**<0.001**	1.06 [0.69; 1.62]	0.803
	MM/SSM	**4.41 [3.26; 5.98]**	**<0.001**	1.17 [0.79; 1.72]	0.442
Clark level	IV/I-II-III	**2.93 [2.15; 4.00]**	**<0.001**	1.11 [0.93; 1.33]	0.685
	V/I-II-III	**4.65 [3.14; 6.90]**	**<0.001**	1.19 [0.92; 1.46]	0.721
	occult/I-II-III	**7.19 [4.23; 12.25]**	**<0.001**	1.17 [0.97; 1.60]	0.920
AJCC 8th edition T category	T1b-T2a/T1a	**2.39 [1.44; 3.94]**	**0.001**	1.53 [0.90; 2.61]	0.118
	T2b-T3a/T1a	**5.13 [3.11; 8.45]**	**<0.001**	**2.32 [1.28; 4.20]**	**0.006**
	T3b-T4a/T1a	**6.52 [4.06; 10.47]**	**<0.001**	**2.82 [1.54; 5.19]**	**0.001**
	T4b/T1a	**14.40 [9.37; 22.12]**	**<0.001**	**5.00 [2.73; 9.18]**	**<0.001**
	T0/T1a	**7.47 [3.41; 16.34]**	**<0.001**	**3.29 [1.33; 8.13]**	**0.010**
Regional lymph node metastasis	yes/no	**5.72 [4.38; 7.47]**	**<0.001**	1.34 [0.81; 2.61]	0.455
Distant metastasis at sites M1a	yes/no	**6.79 [5.22; 8.83]**	**<0.001**	**2.11 [1.53; 2.92]**	**<0.001**
Lung metastasis	yes/no	**8.07 [6.24; 10.45]**	**<0.001**	**2.45 [1.79; 3.36]**	**<0.001**
Non–central nervous system visceral metastases	yes/no	**7.31 [5.65; 9.47]**	**<0.001**	**2.10 [1.53; 2.89]**	**<0.001**

Significant results are in bold. HR [95% CI]—hazard ratio with 95% confidence intervals; LMM—lentigo maligna melanoma; SSM—superficial spreading melanoma; NM—nodular melanoma; MM—unclassified malignant melanoma or no evidence of primary tumor; AJCC—American Joint Committee on Cancer; T—primary tumor; M1a—metastases to skin, soft tissue (including muscle), and/or non-regional lymph nodes.

## Data Availability

The original contributions presented in the study are included in the article/Supplementary Materials; further inquiries can be directed to the corresponding author/s.

## Supplementary Materials

The following supporting information can be downloaded at: https://www.mdpi.com/article/10.3390/cancers17183080/s1, Figure S1. 3-, 5- and 10-year ROC of regional lymph node metastasis nomogram in training cohort (A) and in validation cohort (B), 3-, 5- and 10-year ROC of skin, soft tissue (including muscle) and/or non-regional lymph node metastasis nomogram in training cohort (C) and in validation cohort (D), 3-, 5- and 10-year ROC of lung metastasis nomogram in training cohort (E) and in validation cohort (F), 3-, 5- and 10-year ROC of visceral metastasis nomogram in training cohort (G) and in validation cohort (H), 3-, 5- and 10-year ROC of brain metastasis nomogram in training cohort (I) and in validation cohort (J). Figure S2. 3-, 5- and 10-year calibration plots of regional lymph node metastasis nomogram in training cohort (A) and in validation cohort (B), 3-, 5- and 10-year calibration plots of skin, soft tissue (including muscle) and/or non-regional lymph node metastasis nomogram in training cohort (C) and in validation cohort (D), 3-, 5- and 10-year calibration plots of lung metastasis nomogram in training cohort (E) and in validation cohort (F), 3-, 5- and 10-year calibration plots of visceral metastasis nomogram in training cohort (G) and in validation cohort (H), 3-, 5- and 10-year calibration plots of brain metastasis nomogram in training cohort (I) and in validation cohort (J). Figure S3. 3-, 5- and 10-year DCA of regional lymph node metastasis nomogram in training cohort (A) and in validation cohort (B), 3-, 5- and 10-year DCA of skin, soft tissue (including muscle) and/or non-regional lymph node metastasis nomogram in training cohort (C) and in validation cohort (D), 3-, 5- and 10-year DCA of lung metastasis nomogram in training cohort (E) and in validation cohort (F), 3-, 5- and 10-year DCA of visceral metastasis nomogram in training cohort (G) and in validation cohort (H), 3-, 5- and 10-year DCA of brain metastasis nomogram in training cohort (I) and in validation cohort (J).
