# Supplementary material for: Development of Nomograms to Predict the Probability of Recurrence at Specific Sites in Patients with Cutaneous Melanoma
Source: Cancers (Basel). 2025 Sep 21;17(18):3080. doi: 10.3390/cancers17183080 (PMC12468445; doi:10.3390/cancers17183080)
Supplement: Supplementary file 1 [file cancers-17-03080-s001.zip › cancers-3873640-SI.pdf]

SUPPLEMENTARY MATERIALS

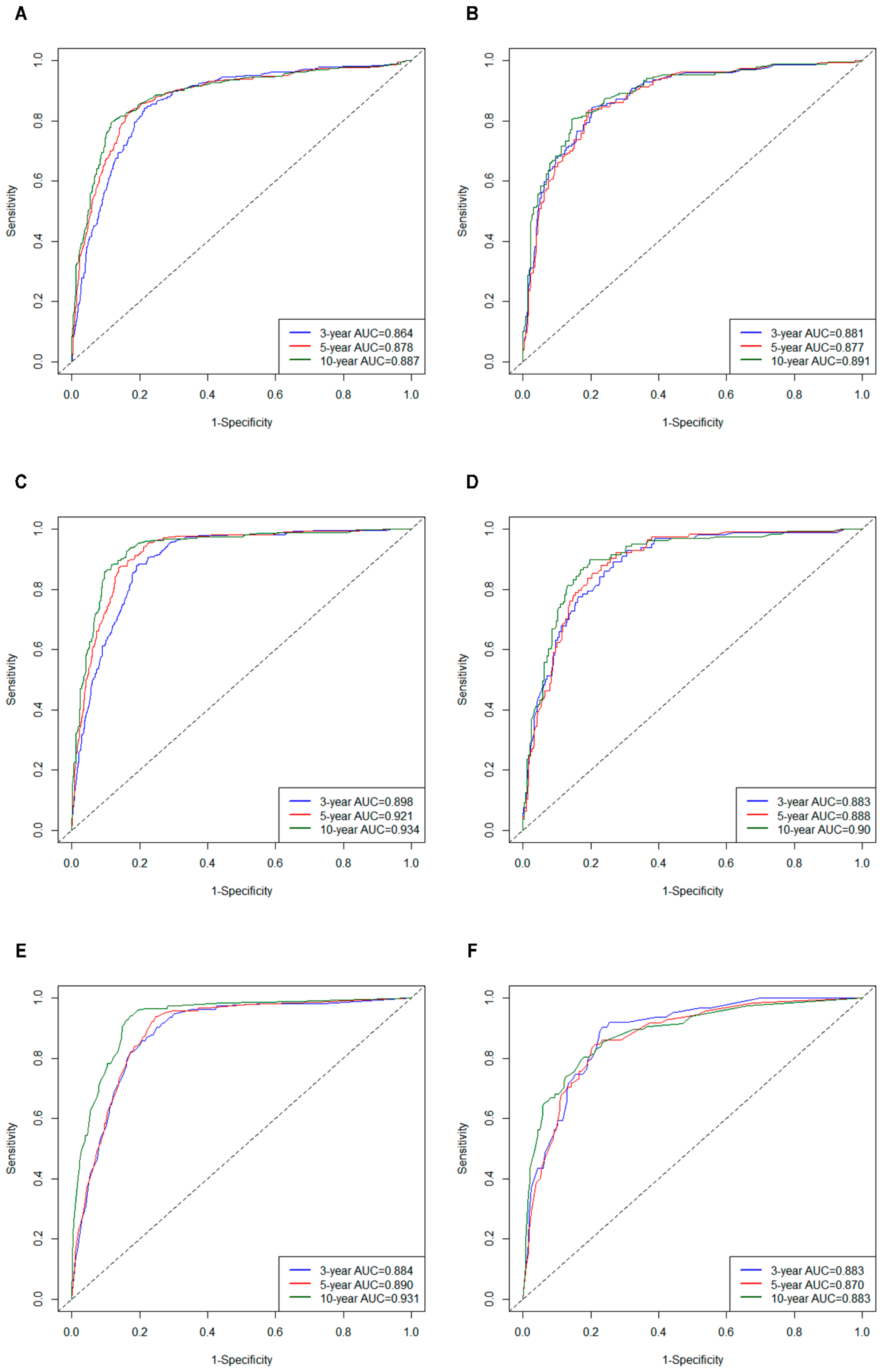

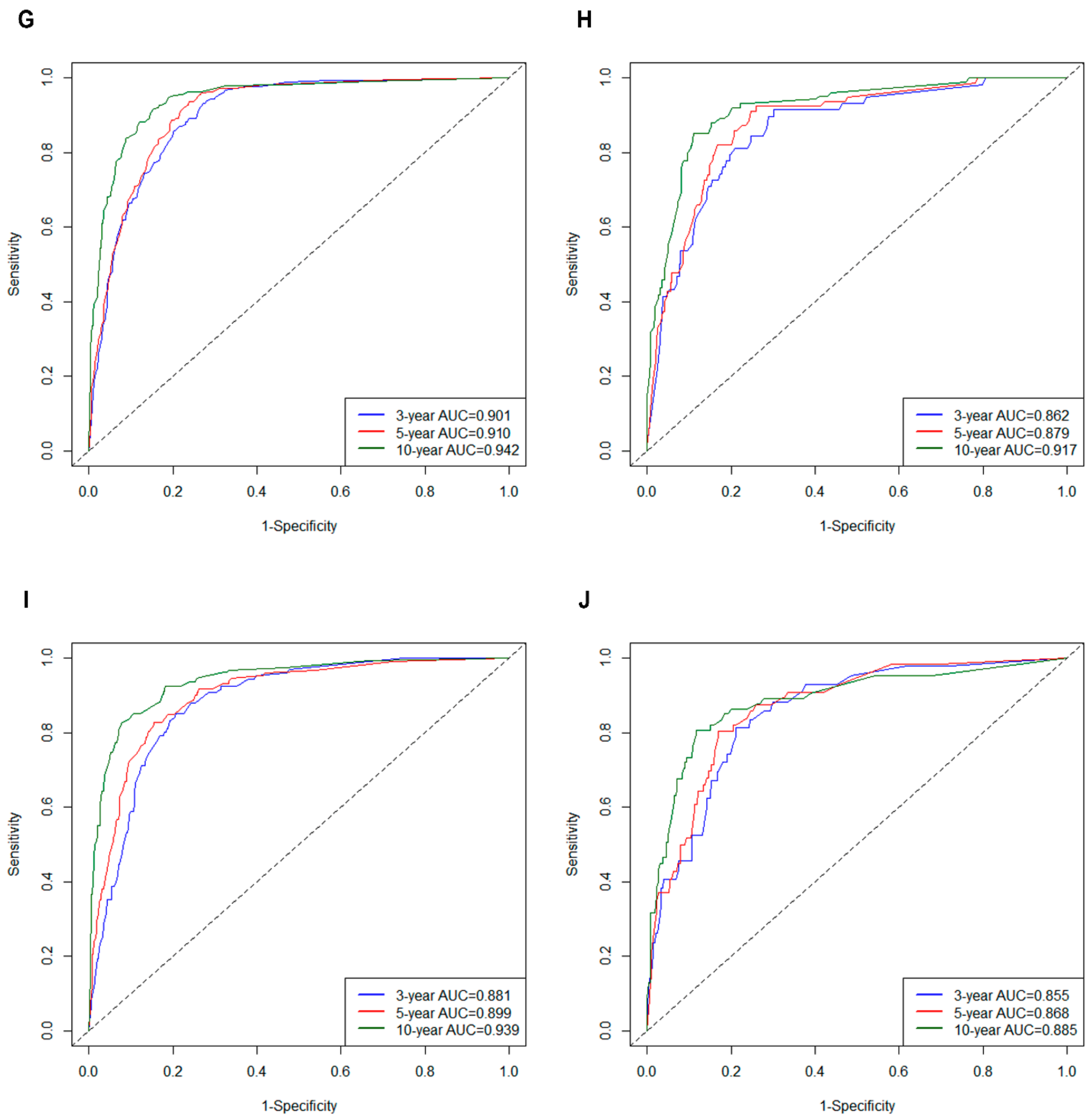

Figure S1.

3-, 5- and 10-year ROC of regional lymph node metastasis nomogram in training cohort (A) and in validation cohort (B), 3-, 5- and 10-year ROC of skin, soft tissue (including muscle) and/or non-regional lymph node metastasis nomogram in training cohort (C) and in validation cohort (D), 3-, 5- and 10-year ROC of lung metastasis nomogram in training cohort (E) and in validation cohort (F), 3-, 5- and 10-year ROC of visceral metastasis nomogram in training cohort (G) and in validation cohort (H), 3-, 5- and 10-year ROC of brain metastasis nomogram in training cohort (I) and in validation cohort (J)

ROC – receiver operating characteristic; AUC – area under the curve

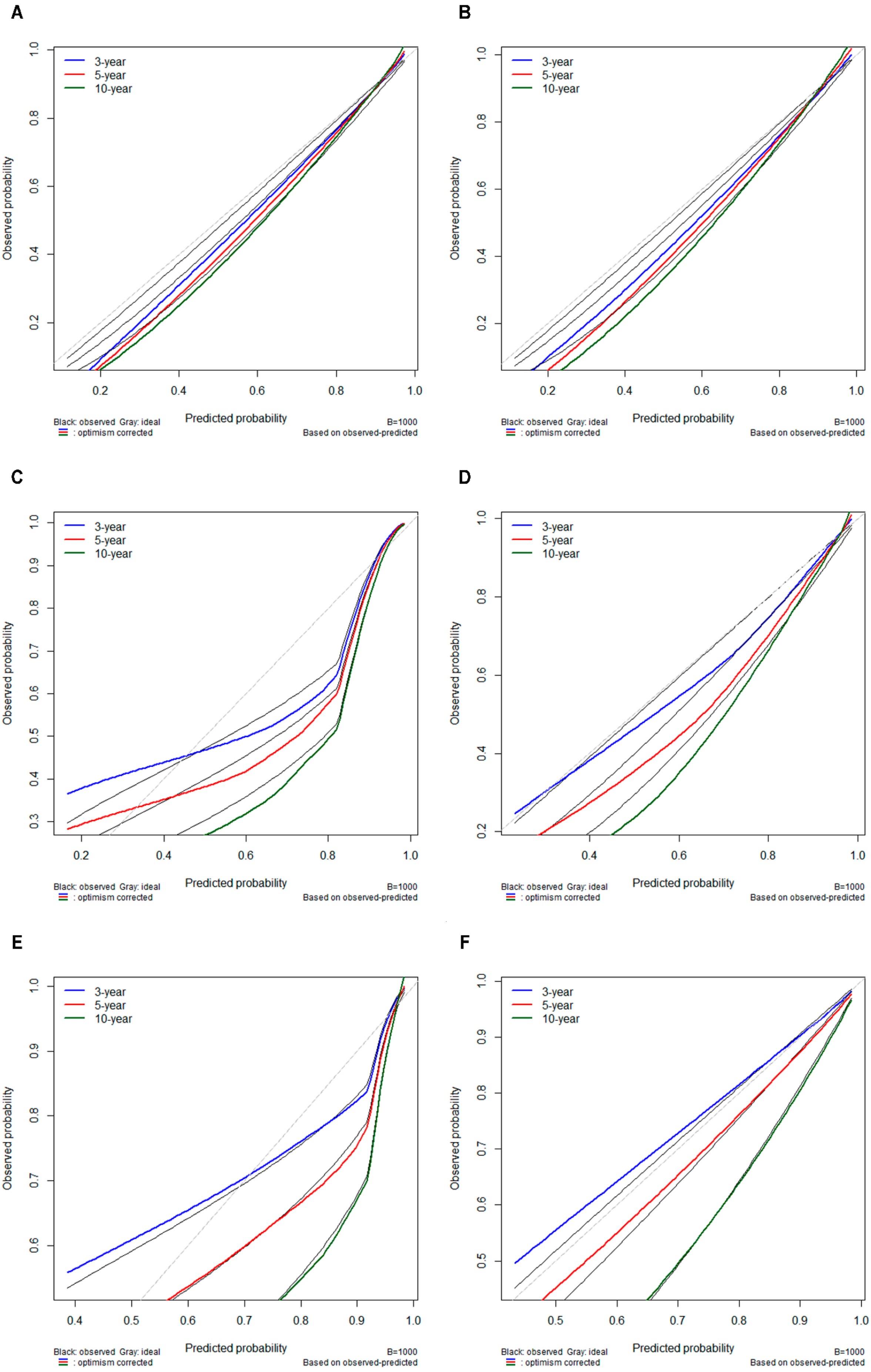
A
B
C
D
E
F
3-year
5-year
10-year
Observed probability
Predicted probability
Black: observed Gray: ideal
: optimism corrected
B=1000
Based on observed-predicted

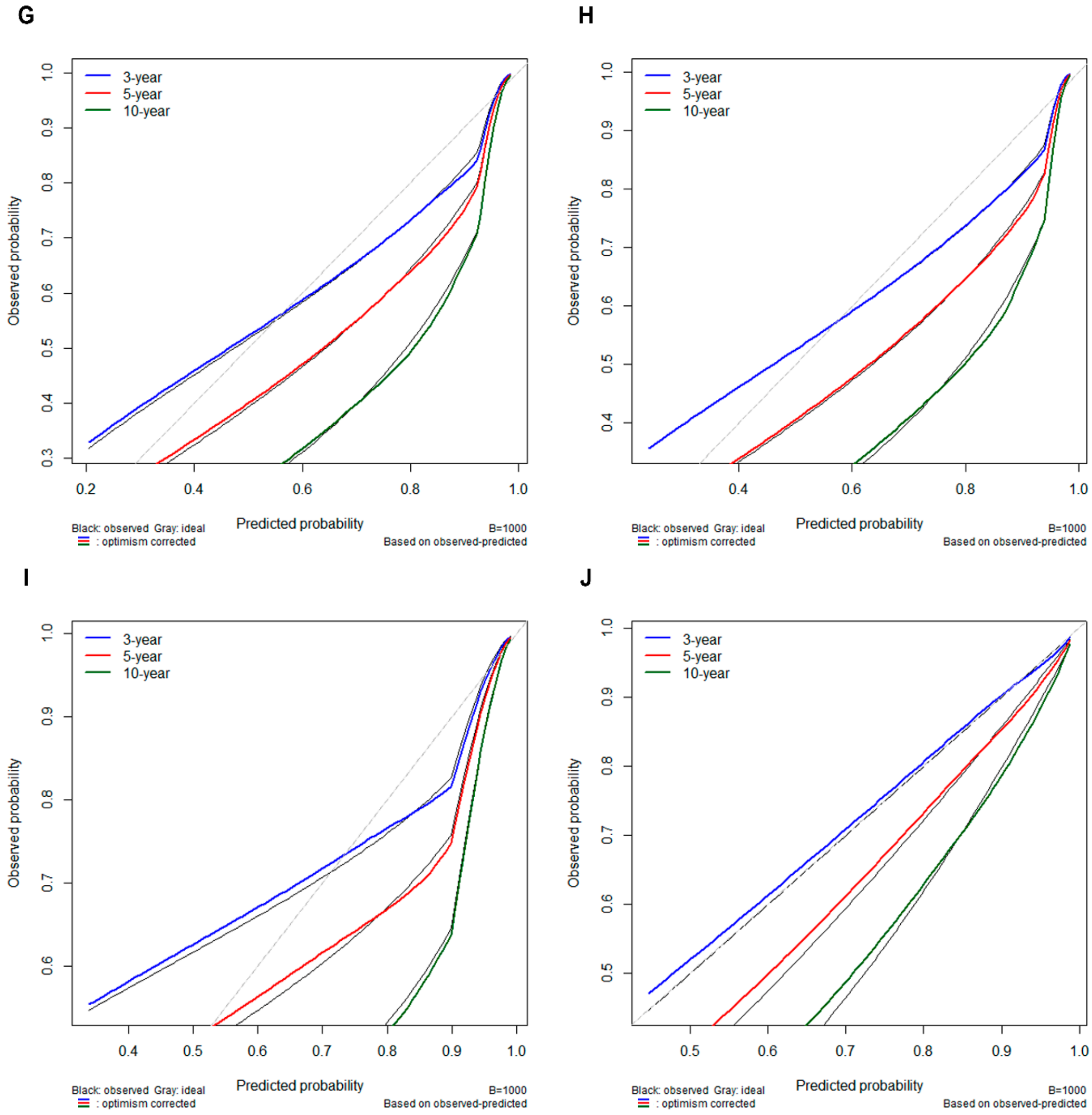

Figure S2.

3-, 5- and 10-year calibration plots of regional lymph node metastasis nomogram in training cohort (A) and in validation cohort (B), 3-, 5- and 10-year calibration plots of skin, soft tissue (including muscle) and/or non-regional lymph node metastasis nomogram in training cohort (C) and in validation cohort (D), 3-, 5- and 10-year calibration plots of lung metastasis nomogram in training cohort (E) and in validation cohort (F), 3-, 5- and 10-year calibration plots of visceral metastasis nomogram in training cohort (G) and in validation cohort (H), 3-, 5- and 10-year calibration plots of brain metastasis nomogram in training cohort (I) and in validation cohort (J)

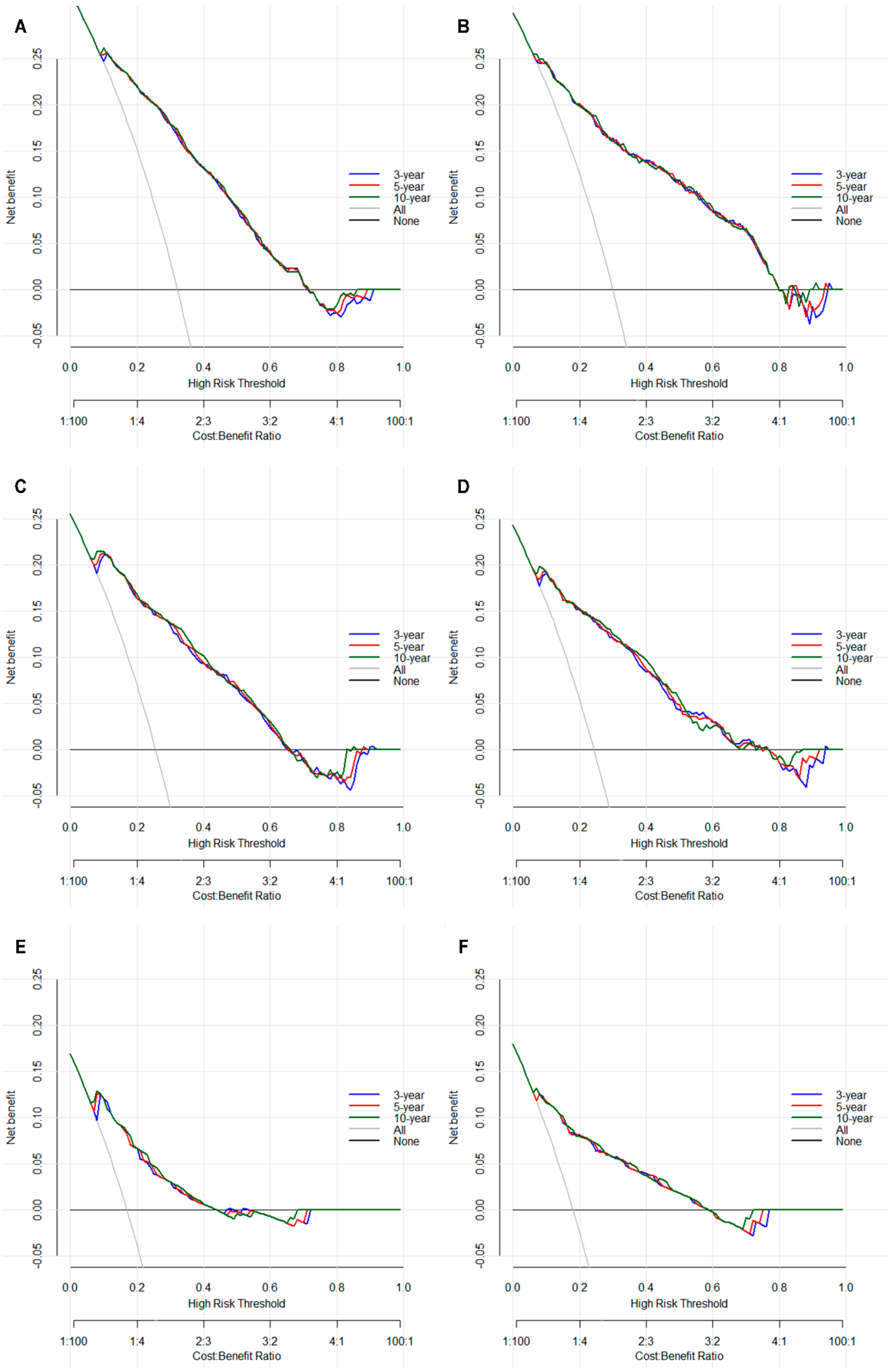
A
B
C
D
E
F
Net benefit
0.25
0.20
0.15
0.10
0.05
0.00
-0.05
3-year
5-year
10-year
All
None
0.0
0.2
0.4
0.6
0.8
1.0
High Risk Threshold
1:100
1:4
2:3
3:2
4:1
100:1
Cost:Benefit Ratio

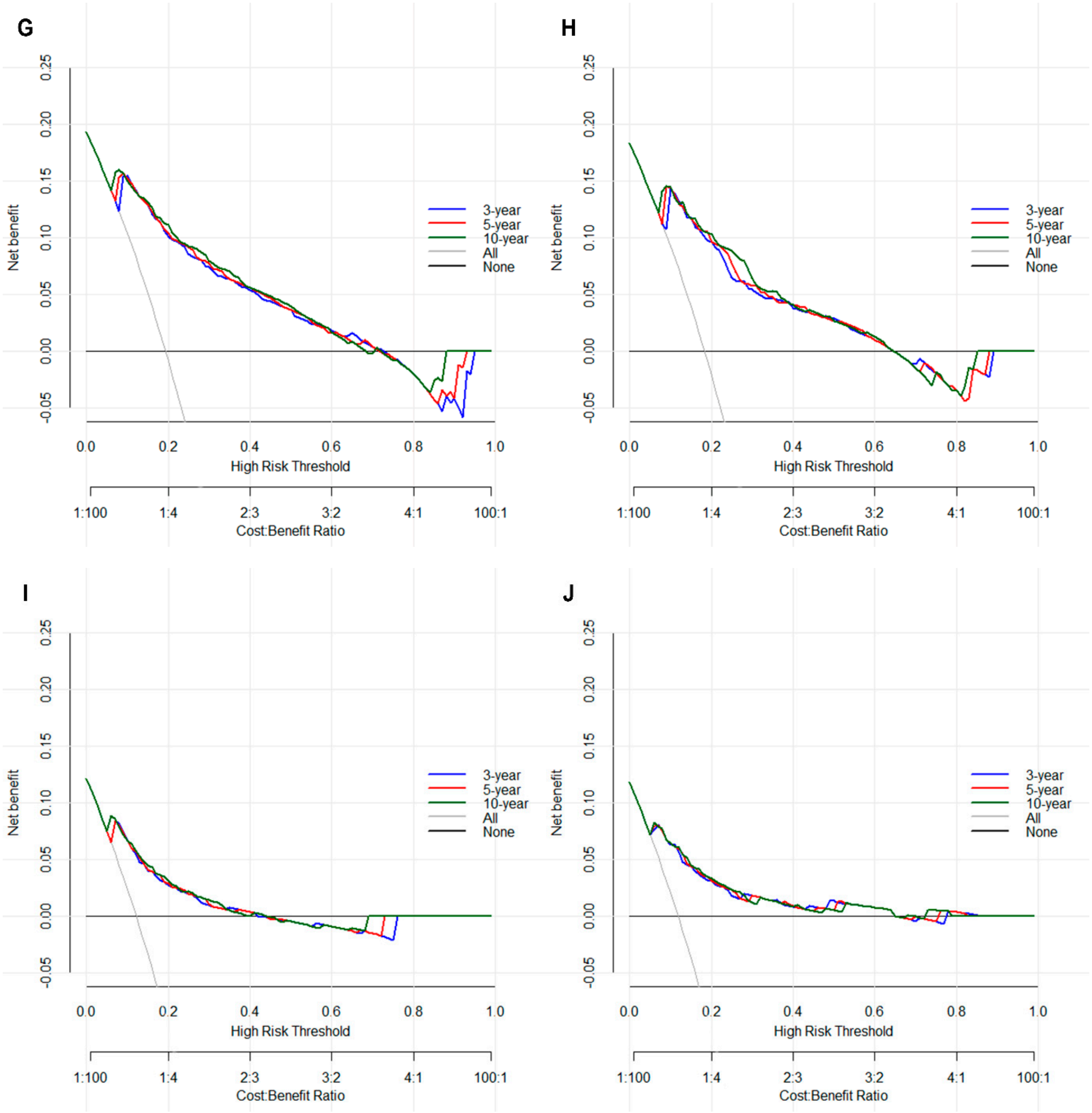

Figure S3.

3-, 5- and 10-year DCA of regional lymph node metastasis nomogram in training cohort (A) and in validation cohort (B), 3-, 5- and 10-year DCA of skin, soft tissue (including muscle) and/or non-regional lymph node metastasis nomogram in training cohort (C) and in validation cohort (D), 3-, 5- and 10-year DCA of lung metastasis nomogram in training cohort (E) and in validation cohort (F), 3-, 5- and 10-year DCA of visceral metastasis nomogram in training cohort (G) and in validation cohort (H), 3-, 5- and 10-year DCA of brain metastasis nomogram in training cohort (I) and in validation cohort (J)

DCA – decision curve analysis
